# Improving Biological Treatment of Real Bilge Wastewater With Zero Valent Iron and Activated Charcoal Addition

**DOI:** 10.3389/fbioe.2020.614510

**Published:** 2020-12-18

**Authors:** Aikaterini A. Mazioti, Gregoris Notarides, Giannis Symeou, Ioannis Vyrides

**Affiliations:** Environmental Engineering Laboratory, Department of Chemical Engineering, Cyprus University of Technology, Limassol, Cyprus

**Keywords:** bilge wastewater, anaerobic granular sludge, activated charcoal, methane, *Acetobacterium*, aerobic biomass

## Abstract

From the ships engine rooms a recalcitrant wastewater is produced called “bilge” which contains oil, metal working fluids, surfactants, and salinity. This study investigated the treatment of real bilge wastewater in short experiments using the following processes: (i) anaerobic digestion with granular sludge and ZVI addition for enhancement of methane production, (ii) activated charcoal addition to biological treatment (aerobic and anaerobic) for Chemical Oxygen Demand (COD) significant reduction and (iii) combination of ZVI and anaerobic charcoal addition for high performance treatment. The addition of ZVI in anaerobic sludge resulted in higher performance mostly in cumulative CH_4_ production. The microbial profile of anaerobic granular sludge exposed to ZVI was determined and *Acetobacterium* and *Arcobacter* were the most dominant bacteria genera. Activated charcoal achieved higher COD removal, compared to biological degradation (aerobic and anaerobic). The combination of the two mechanisms, activated charcoal and biomass, had higher COD removal only for aerobic biomass. The combination of ZVI and activated charcoal to anaerobic digestion resulted in higher CH_4_ production and significant COD removal in short contact time.

## Introduction

Bilge wastewater is generated in the ships engine room and is stored in the lowest part of the ship called bilge tank (Uma and Gandhimathi, 2019). Bilge is a recalcitrant wastewater that contains diesel oil, soluble oil, metal working fluids, surfactants and has high salinity (15–30 g L^–1^). McLaughlin et al. (2014) reported that bilge wastewater contains hydrocarbons such as toluene, benzene, butylbenzene, ethylbenzene, acenaphthene, phenanthrene, pyrene, fluorene, metals, and substantial concentrations of detergents and solvents. The high concentration of surfactants causes the solubilization of high concentration of oil compounds in bilge wastewater and this contributes to high chemical oxygen demand (COD) (Vyrides et al., 2018b). In addition, according to International Maritime Organization (IMO) regulations (MARPOL 73/78) and the European directive 2000/59/EC bilge wastewater cannot be discharged to environment and should be properly treated enroute or to be deposited at reception facilities on land (Julian, 2000; Vyrides et al., 2018b). Treatment processes include physicochemical methods, such as oil separation techniques, coagulation, chemical oxidation, membrane filtration and others, as well as biological treatment methods (Varjani et al., 2020).

Up to date several studies examined the biological treatment of bilge wastewater using aerobic mix consortia. Specifically, Vyrides et al. (2018b) found approximately 60% Chemical Oxygen Demand (COD) removal from real bilge wastewater in a pilot (200 L) Moving Bed Biofilm Reactor (MBBR) system. Nisenbaum et al. (2020) used an enriched aerobic microbial consortium to treat bilge wastewater and found 66.65, 72.33, and 97.76% removal of total petroleum hydrocarbons, aromatics and n-alkanes, respectively. Hwang et al. (2019) used single chamber microbial fuel cells (MFCs) for bilge wastewater biodegradation and concurrent electricity generation. In this system, the COD removal was greatly increased with the addition of anionic surfactant (SDS).

Regarding the anaerobic treatment of bilge water, Emadian et al. (2015) reported 75% COD removal of low strength bilge water whereas Vyrides et al. (2018b) in a batch test pointed out 28% of COD removal from real bilge wastewater. As a strategy to treat recalcitrant wastewater and enhance methane production several studies examined chemicals addition to the process. A recent study examined CaO_2_ pretreatment of waste activaded sludge in order to degrade refractory compounds such as humus and lignocelluloses, increasing importantly methane production during anaerobic digestion (Wang et al., 2019). Other researchers added zero valent iron directly in the anaerobic digestion process; Liu et al. (2011) for dye wastewater, Wang et al. (2017) for Fischer-Tropsch (FT) wastewater, Xu et al. (2020) for coking wastewater and Pan et al. (2019) for tetracycline biodegradation. The addition of ZVI in the anaerobic digestion results in the abiotic H_2_ production due to anaerobic oxidation of ZVI. The generated H_2_ is utilized by hydrogenotrophic methanogens along with CO_2_ and therefore increase CH_4_ in the system. In addition the ZVI anaerobic oxidation provides alkalinity in the system and due to the enhancement of hydrogen utilizing microorganisms can contribute to propionic acid utilization.

Regarding adsorption method so far only Vlaev et al. (2011) investigated the bilge wastewater adsorption using sing rice husks ash. The results showed that rice husks ash had high adsorption capacity for hydrocarbon removal for bilge water. In addition, the final product was characterized with high calorific value and was proposed to be used as a feedstock in incinerators, industrial ovens or steam generators.

The aim of this study was to improve biological treatment of bilge wastewater using advanced processes. Biological treatment was chosen as a relatively inexpensive and easy to apply method. For this reason the following conditions were examined under batch mode experimental set up and at small laboratory scale.

(A) Firstly, anaerobic digestion of bilge wastewater was examined as an initial biological treatment step for decreasing high organic load and the addition of ZVI was evaluated as a strategy to increase methane production. The microbial profile under these conditions was studied using next generation sequencing.
(B) Secondly, a purification step was tested, combining activated charcoal adsorption and biological activity. Both aerobic and anaerobic conditions were tested in this case in order to determine the more efficient for organic load decrease.
(C) Finally, both treatment steps were combined in order to evaluate treatment performance and reduce treatment time. In this case anaerobic digestion was examined with the simultaneous addition of (i) ZVI; for methane enhancement, and (ii) activated charcoal; for organic load decrease.

To the author’s knowledge, this is the first study examining ZVI addition for enhancement of real bilge wastewater anaerobic treatment, and the first to combine this approach with activated charcoal as a second individual treatment step or as a combination of processes.

## Materials and Methods

### Anaerobic Digestion of Bilge Wastewater

For all anaerobic experiments, sealed glass serum bottles were used and were incubated at 37°C. Anaerobic granular sludge was used at a concentration of 4% w/v, collected from a mesophilic upflow anaerobic sludge blanket reactor (UASB) that was treating dairy wastewater (Charalambides Christis Ltd, Limassol, Cyprus), operated at pH 6.8–7.3 (as reported by Samanides et al., 2020). Raw bilge wastewater was used, collected by a company treating this wastewater type (Ecofuel Ltd, Zygi, Cyprus), the wastewater characteristics are described in detail in a previous study by Vyrides et al. (2018b). A mineral medium as described by Angelidaki et al. (2009) was added to create the same initial concentration in all experiments. Raw bilge wastewater was enriched with the following stock solutions (chemicals given are concentrations in g L^–1^ in distilled water) Solution A: NH_4_Cl, 100; NaCl, 10; MgCl_2_6H_2_O, 10; CaCl_2_2H_2_O, 5, Solution B: K_2_HPO_4_3H_2_O, 200. To 993 ml of raw bilge wastewater the following volume of each stock solution was added: (Solution A) 5 ml, (Solution B) 2 ml as well as 3 g of NaHCO_3_. As a last step, prior to incubation, the serum bottles were sealed with rubber septa and a screw cap and the headspace was flushed with CO_2_ (99.99% purity) for 1 min for the creation of anaerobic conditions.

Preliminary investigation included the test of three dilution rates for the treatment of bilge wastewater of low initial COD with anaerobic digestion. The following dilution factors were applied to the wastewater: 1:1, 1:2, and 1:4, leading to wastewater concentration of 100, 50, and 25% in each case. Serum bottles of 125 ml were used with a working volume of 70 ml. Treatment time was 82 days.

Monitoring of the anaerobic digestion process included the determination of biogas volume production as well as gas composition of the headspace. For this reason a gas chromatograph, coupled with a thermal conductivity detector (GC-TCD, Agilent technologies 7820A GC system, Wilmington, DE) was used, according to the method described by Vyrides et al. (2018b). Furthermore, the Chemical Oxygen Demand (COD) value of the wastewater was measured at the beginning, during and at the end of the experiments. For COD quantification a modified colorimetric method was used (absorbance quantification with HACH, DR1900 spectrophotometer), according to Freire and Sant’Anna (1998) and Vyrides and Stuckey (2009) in order to take into consideration the oxidation of chloride ions present in bilge wastewater due to high salinity.

### ZVI Batch Experiments

For the examination of more realistic industrial conditions, the enhancement of anaerobic digestion of high organic load bilge wastewater with the use of Zero-Valent-Iron (ZVI) was tested as a technique to facilitate microbial activity via H_2_ production. Powder ZVI (Merck, Iron for analysis reduced, particle size 10 μm, CAS-No: 7439-89-6) was used at two concentration levels (10, 25 g L^–1^) in a small scale batch experiment (125 ml glass serum bottles with 70 ml working volume) and in a second experimental cycle the periodic addition of ZVI was tested under semi batch conditions in a larger scale experiment (500 ml glass bottles with 300 ml working volume). Bilge wastewater, granular anaerobic sludge and anaerobic conditions were obtained and handled as described in section “Anaerobic Digestion of Bilge Wastewater.” COD and gas composition were monitored over time in order to evaluate digestion performance. The microbial profile (bacteria and archea) of the granular sludge was evaluated after 47 days of operation under semi batch conditions (larger scale experiment). Approximately 180–220 mg of biomass were collected from each bioreactor. Then the total genomic DNA was extracted through the NucleoSpin DNA stool (Macherey-Nagel, Germany) and was sent to DNASense Company (Denmark) for sequencing.

### Activated Charcoal Batch Experiments

Activated charcoal was tested conjointly with microorganisms for further reduction of organic load in bilge wastewater. Aerobic and anaerobic experiments were conducted with medium initial COD level and under batch mode in sealed glass serum bottles (125 ml) with 70 ml working volume and 55 ml of headspace. Activated charcoal (AC) (Fluka, CAS-No: 7440-44-0) was used at the concentration level of 3 g L^–1^ whereas optimum performance was observed after preliminary experiments.

For anaerobic treatment, the set up of the experiments was conducted as described in section “Anaerobic Digestion of Bilge Wastewater.” For aerobic treatment, a liquid mixed bacteria culture, obtained by enrichment steps (3 months), was used with the addition of 4% v/v in the serum bottle. For the examination of aerobic treatment performance, COD was monitored and UV-VIS spectra of the effluent was examined using a benchtop spectrophotometer (Jenway 7315 UV/Visible spectrophotometer).

### Combined ZVI and Activated Charcoal Batch Experiment

The combination of the techniques described in section “ZVI Batch Experiments” and section “Activated Charcoal Batch Experiments” were applied in order to test the parallel reduction of high organic load in combination with high CH_4_ production. Sealed glass serum bottles (125 ml) with 70 ml working volume and 55 ml of headspace were used and anaerobic granular sludge was added at a concentration of 4% w/v. Anaerobic digestion of bilge wastewater of high initial COD was examined (i) with granular sludge and with (ii) granular sludge with ZVI (10 g L^–1^) and activated charcoal (3 g L^–1^) addition at the beginning of the experiment. Samples were created in triplicates and monitored over 15 days, as described in section “Anaerobic Digestion of Bilge Wastewater.”

## Results

### Treatment of Bilge Wastewater Using Anaerobic Granular Sludge With and Without Zero Valent Iron Addition

**FIGURE 1:**
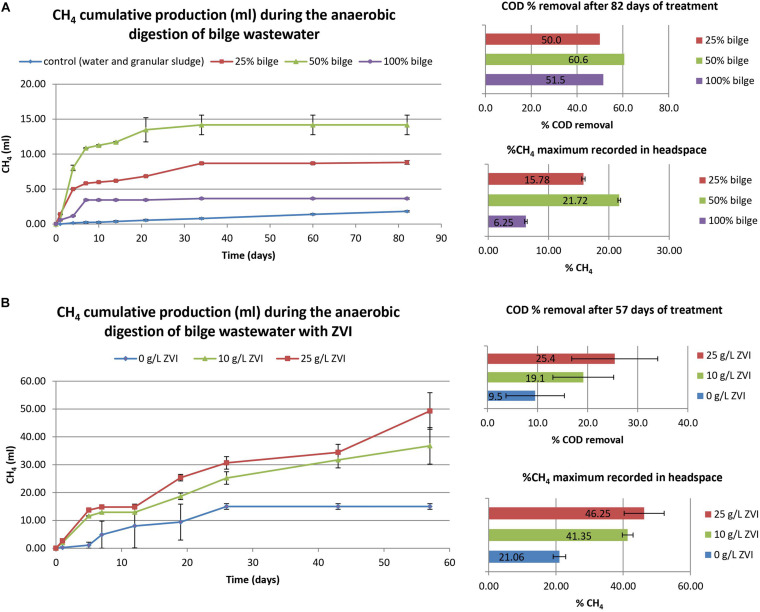
Performance of anaerobic digestion of bilge wastewater with the use of granular sludge **(A)** under different dilution factors (100, 50, and 25% concentration of bilge) and low initial COD (2200, 1100, and 550 mg L^–1^) and **(B)** with the addition of powder ZVI (25, 10, and 0 g L^–1^) and with a high initial COD (5150 mg L^–1^).

Figure 1A points out that the exposure of anaerobic granular sludge to undiluted real bilge wastewater (initial COD 2200 mg L^–1^) resulted in relatively low methane production over a period of 82 days. The dilution of the wastewater caused higher methane production, probably as a result of toxicity decrease. The COD removal after 82 days varied from 50 to 60% under these low initial COD conditions.

As a strategy to alleviate bilge inhibition to anaerobic granular sludge, zero valent iron was added at 10 and 25 g ZVI L^–1^. As shown in Figure 1B, addition of ZVI generated higher amount of CH_4_ compared to anaerobic granular sludge without ZVI. The COD removal under these conditions was low (Figure 1B), however, the COD removal in the reactors containing ZVI was slightly higher compared to the reactors with anaerobic granular sludge free of ZVI. Noteworthy, the initial COD was 5150 mg L^–1^ which was more than double than the experiment related with Figure 1A. It is likely that the higher COD in the experiment related with Figure 1B has contributed to higher toxicity to anaerobic granular sludge and therefore the anaerobic granular sludge (with no ZVI) resulted in negligible COD removal. Part of the higher CH_4_ composition (Figure 1B) in anaerobic granular sludge with ZVI could be due to H_2_ production due to anaerobic aquatic zero valent iron oxidation followed by the H_2_ and CO_2_ utilization by hydrogenotrophic methanogens (Vyrides et al., 2018a; Menikea et al., 2020).

Based on the positive findings regarding the ZVI addition a larger scale (500 ml glass bottles with 300 ml working volume) experiment took place as descripted in section “Materials and Methods.” As shown at Table 1, at the beginning of the experiment the anaerobic granular sludge with 10 g L^–1^ ZVI resulted in dramatically higher methane production (55.5 ml CH_4_ at day 7) compared to anaerobic granular sludge free of ZVI (8.5 ml CH_4_ at day 7). This trend was continued through the experiment and higher performance was found for the bioreactor with 10 g L^–1^, followed by the bioreactor with 2 g L^–1^ initial ZVI concentration (were ZVI 2 g L^–1^ was periodically added). The lower performance was found in the anaerobic granular sludge free of ZVI (Table 1). This difference was also more profound in the CH_4_ composition under these conditions.

**TABLE 1:** Performance and operational parameters of ZVI batch experiment (large scale) for bilge wastewater anaerobic digestion.

Large Scale semi batch anaerobic digestion reactor performance (500 ml reactor volume with 300 ml working volume), *T* = 37°C													

Operation day	1	7	8	14	21	22	28	35	37	43	47	48	77
**CONTROL**													
CH_4_ % in headspace	0.2	3.8		27.2	15.1		15.0	9.0		0.6	0.9		0.8
CH_4_ cumulative production (ml)	0.6	8.5		88.9	136.7		147.2	154.8		156.1	156.7		156.7
pH adjustment			✓			✓			✓			✓	
CO_2_ flush			✓			✓			✓			✓	
**EXP 1 (10 g/L ZVI)**													
CH_4_ % in headspace	3.4	27.5		9.1	14.2		34.7	40.1		14.9	22.1		33.2
CH_4_ cumulative production (ml)	7.3	55.5		73.6	114.7		157.7	182.9		212.8	227.2		249.3
pH adjustment			✓			✓			✓			✓	
CO_2_ flush			✓			✓			✓			✓	
**EXP 2 (2 g/L ZVI)**													
CH_4_ % in headspace	2.3	19.4		9.4	11.3		29.0	41.2		16.6	23.0		32.0
CH_4_ cumulative production (ml)	5.4	39.6		60.1	93.4		128.8	153.1		186.2	199.0		217.2
pH adjustment			✓			✓			✓			✓	
CO_2_ flush			✓			✓			✓			✓	
ZVI addition (2 g/L)			✓			✓			✓			✓	

**FIGURE 2:**
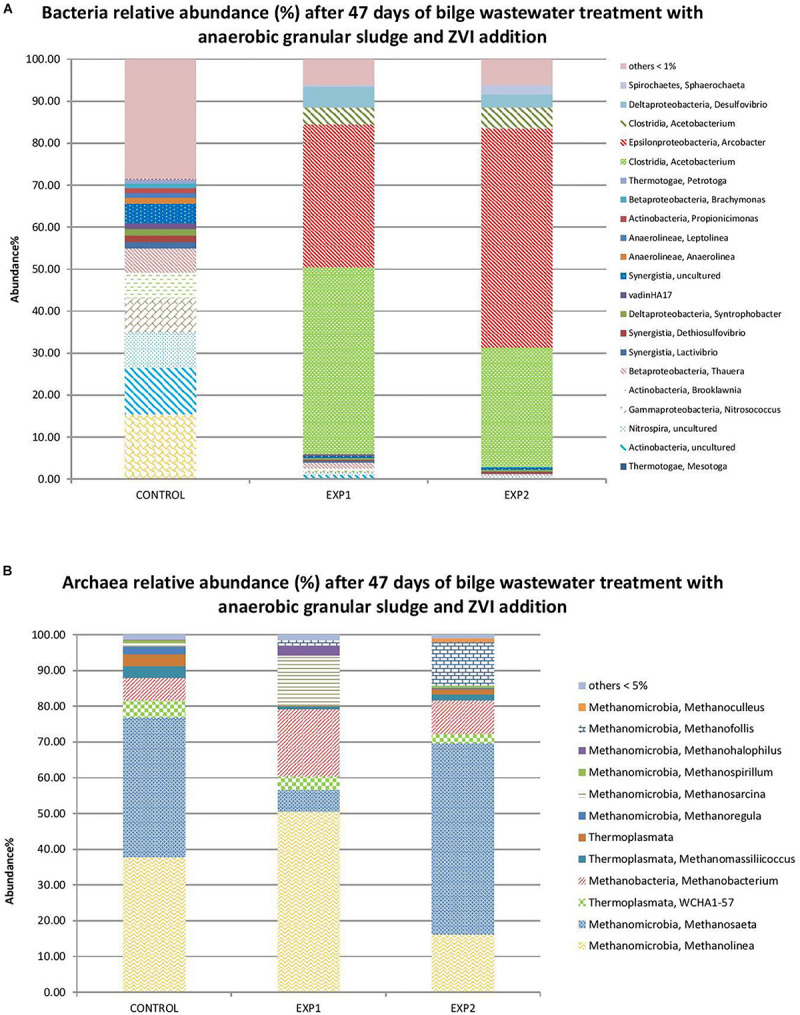
Microbial profile of anaerobic granular sludge after 47 days of exposure to bilge wastewater (CONTROL), with 10 g L^–1^ powder ZVI addition at *t* = 0 (EXP 1) and 2 g L^–1^ powder ZVI addition at *t* = 0 (EXP 2). Relative abundance % is illustrated for populations of **(A)** bacteria and **(B)** archaea.

The relative abundance of the archaeal 16S rRNA gene at the genus levels is shown in Figure 2A. Samples were withdrawn from bioreactors on day 47. The most dominant genus in anaerobic sludge with ZVI were *Acetobacterium* and *Arcobacter*. However, these genera were negligible in anaerobic granular sludge without ZVI (Figure 2A). *Acetobacterium* are homoacetogens and can convert H_2_ and CO_2_ to acetic acid; they were stimulated due to the presence of ZVI and the release of H_2_ (Menikea et al., 2020). The *Arcobacter* genus includes a diverse assemblage of species which are obligate and facultative chemolithoautotrophs as well as heterotrophs. *Arcobacter* spp. have been enriched in engineered systems containing high levels of sulfide and high levels of organic matter and benefit from the transfer of organic matter and hydrogen (Callbeck et al., 2019).

*Methanosaeta* sp. (acetoclastic methanogens) have been proposed as one of the primary microbial groups responsible for methanogenic granule formation and this explains its high abundance in all samples; however, a higher relative abundance was found in anaerobic granular sludge that was exposed to 10 g L^–1^ of ZVI (Figure 2). These findings are in line with the study of Vyrides et al. (2018a) which found higher relative abundance in anaerobic granular sludge exposed to ZVI. *Methanolinea*, a hydrogenotrophic methanogen, was the dominant genus (∼50%) in the anaerobic granular sludge where 2 g ZVI L^–1^ was periodically added in the bioreactor (Table 1 and Figure 2B). Finally, another hydrogenotrophic methanogen, *Methanobacterium*, was found at a higher relative abundance in anaerobic granular sludge exposed to ZVI compare to anaerobic granular sludge free of ZVI (Figure 2B).

### Activated Charcoal

**FIGURE 3:**
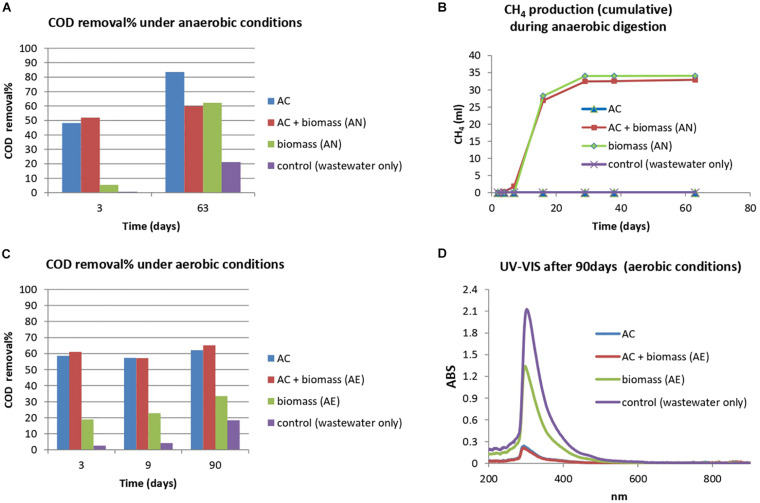
Treatment of bilge wastewater with the use of anaerobic/aerobic biomass and activated charcoal (AC), COD reduction of bilge wastewater (initial COD: 2750 mg L^–1^) **(A)** with the addition of (i) activated charcoal (AC), (ii) AC and anaerobic biomass (granular sludge), (iii) anaerobic biomass (granular sludge), (iv) control (bilge wastewater only), and **(B)** CH_4_ cumulative production (ml) under anaerobic conditions, **(C)** COD reduction of bilge wastewater (initial COD: 3470 mg L^–1^) with the addition of (i) activated charcoal (AC), (ii) AC and aerobic biomass, (iii) aerobic biomass, (iv) control (bilge wastewater only), **(D)** UV-VIS absorbance values of the treated effluent after 90 days with aerobic conditions.

As observed in preliminary and ZVI anaerobic experiments, the COD removal from bilge wastewater was relatively low, requiring considerable amount of time, probably due to the toxicity of the wastewater toward anaerobic granular sludge. As a strategy to overcome this, the treatment of bilge wastewater in a system of activated charcoal and anaerobic granular sludge was examined. At an initial COD level of 2750 mg L^–1^, with the presence of activated charcoal in the anaerobic granular sludge reactor, 52 and 60% COD removal rates were achieved after 3 and 63 days of exposure, respectively. Under the same conditions, activated charcoal without anaerobic granular sludge, removed 48 and 84% of the COD load (after 3 and 63 days, respectively). The lower COD removal observed after 63 days of treatment when both activated charcoal and granular sludge were present could be attributed to the decrease of sorption capacity because of VFAs production and adsorption onto activated charcoal after the first days of anaerobic digestion. This trend was observed in a similar study by Jiang et al. (2021) where VFAs where monitored and found to be of lower concentration when activated carbon was present in the anaerobic digestion process. Anaerobic granular sludge without addition of activated charcoal pointed out low COD removal on day 3 and then the removal increased to 62% on day 63. The methane production of anaerobic granular sludge during bilge treatment with and without activated charcoal was similar (Figure 3B). It is highly probable that in the system of anaerobic granular sludge where activated charcoal is present, COD is removed through adsorption on activated charcoal during the first days of treatment and then part of the organics are biodegraded by anaerobic granular sludge (Figure 3A).

A similar experiment was conducted testing the addition of activated charcoal under aerobic conditions and with the addition of an aerobic mixed culture instead of anaerobic granular sludge (Figure 3C). After 3 days of treatment, the COD removal (initial COD: 3470 mg L^–1^) achieved by aerobic biomass with activated charcoal and activated charcoal exclusively was 59 and 61%, respectively. The COD removal slightly increased over time, reaching 62 and 65% removal after 90 days of treatment (for aerobic biomass with activated charcoal and activated charcoal, respectively) On the other hand, aerobic biomass without any activated charcoal pointed out the lower COD removal (19% on day 3 and 33% on day 90). It is highly probable that the contribution of biomass is not significant due to the rapid adsorption of easily biodegradable compounds (low molecular weight organic compounds) on activated charcoal, creating a hostile environment for microorganisms whereas, compounds hard to biodegrade are left in the liquid phase increasing toxicity. The UV-VIS spectra of treated effluent (after 90 days of treatment) revealed the significant impact of activated charcoal addition (Figure 3D). The compounds detected at around 290 nm were substantially removed in the reactors containing activated charcoal and remained present in the reactors free of activated charcoal.

### Effect of ZVI and Activated Charcoal Combined Addition

The performance of anaerobic digestion of bilge wastewater with granular sludge was evaluated over the same process with the addition of ZVI and activated charcoal. The addition of both compounds simultaneously had a positive effect on the treatment performance. Over 15 days of treatment the conventional anaerobic digestion reduced less than 5% of the initial COD of the wastewater while only 2.5 ± 0.1 ml of CH_4_ were produced. On the other hand, with the addition of ZVI and activated charcoal almost 50% of initial COD was reduced in 15 days, while the CH_4_ production reached 44.3 ± 3.4 ml and the CH_4_ percentage in the biogas reached 76.3 ± 2.5% (see Supplementary Material).

## Discussion

The treatment of real bilge wastewater with a high variability in the organic content was examined in short experiments using (i) anaerobic digestion with granular sludge and ZVI addition, (ii) biological treatment (aerobic and anaerobic) with activated charcoal addition, and (iii) combination of ZVI and activated charcoal addition in anaerobic digestion. It was found that undiluted bilge wastewater (initial COD 2200 mg COD L^–1^) showed little cumulative CH_4_ production whereas dilution leading to 50 and 25% concentration of the wastewater resulted in higher cumulative CH_4_. Under these conditions the COD removal was between 50 to 60% after 82 days of treatment. In another experiment where the initial COD was 5150 mg L^–1^ anaerobic granular sludge removed negligible percentage of COD and this showed that the higher the initial COD the harder for the microorganisms to efficiently degrade the wastewater, probably due to higher toxicity toward anaerobic granular sludge. At initial COD 5150 mg L^–1^ the anaerobic granular sludge with ZVI pointed out higher performance even though the difference with anaerobic granular sludge without ZVI was more profound in CH_4_ than in the COD removal. The higher CH_4_ could be due to abiotic anaerobic ZVI oxidation and the released H_2_ that was then utilized by hydrogenotrophic microorganisms. The higher performance of anaerobic granular sludge with ZVI was also found at a larger scale experiment (500 ml glass bottles with 300 ml working volume) whereas anaerobic granular sludge with ZVI 10 g L^–1^ resulted in the highest performance followed by anaerobic granular sludge where ZVI 2 g L^–1^ was periodically added. As previously described (section “Treatment of Bilge Wastewater Using Anaerobic Granular Sludge With and Without Zero Valent Iron Addition”), the H_2_ produced is probably the main mechanism increasing the methane production. Regarding the two addition methods tested (10 g L^–1^ initial addition or progressively addition of 2 g L^–1^ each time) it seems that with a low concentration periodical addition of ZVI, H_2_ is released at a lower rate, rendering the process less effective (Vyrides et al., 2018a). The lowest performance was identified for anaerobic granular sludge without any addition of ZVI in terms of total CH_4_ production and CH_4_ concentration in biogas composition. The bacteria at genus level with the higher relative abundance in anaerobic granular sludge with ZVI were *Acetobacterium* and *Arcobacter*, which can utilize H_2_ along with CO_2_ or organic matter (Callbeck et al., 2019). *Methanosaeta* sp. (acetoclastic methanogens) found in high abundance in all anaerobic granules probably due to its role in granule formation. On the other hand, Methanobacterium (hydrogenotrophic methanogen), was found at a higher relative abundance in anaerobic granular sludge exposed to ZVI compare to anaerobic granular sludge free of ZVI most likely due to the presence of H_2_ due to anaerobic ZVI oxidation.

The addition of activated charcoal in anaerobic granular sludge resulted in fast COD removal after 3 days, however, the COD removal has slightly increased over time. Interestingly, the cumulative CH_4_ was the same for both conditions (anaerobic sludge with activated charcoal and without) and this indicate that activate charcoal contributes mostly by organics adsorption and has not enhanced further the organic biodegradation by anaerobic granular sludge. The same trend was also found for aerobic biomass with activated charcoal which shows approximately 60% COD removal after 3 days whereas aerobic biomass only resulted in 19% COD removal during the same contact time.

These results point out that for rapid COD removal a physicochemical process (activated charcoal) is needed and that the biological process (anaerobic and aerobic) is slow due to toxicity of bilge toward biomass and/or due to recalcitrant organics compounds in bilge wastewater. The integration of activated charcoal in biomass (aerobic or anaerobic) resulted in high COD removal in the first days and then the COD removal was stabilize; however, more research can be done to examine its effect over longer time period. It is probable that the adsorption capacity of activated charcoal is high at the beginning of the treatment, leading to fast COD deterioration, but as activated charcoal reaches saturation the removal rate gets stable (Jiang et al., 2021).

The combination of ZVI and activated charcoal addition had a positive effect on the anaerobic digestion process. Both COD decrease and CH_4_ production were achieved at a relatively low contact time. Further research could focus on the underlying mechanisms of this treatment scheme, while larger scale experiments could determine the feasibility of the method application.

To summarize, greater bioremediation performance, in terms of COD removal, was observed when activated charcoal was added to the biological process. Additionally, the amount and quality of biogas produced during the anaerobic digestion of bilge wastewater can be increased via ZVI addition to the wastewater. The independent use of the two techniques could be used for efficient treatment but would increase treatment time. The combination of both approaches provided promising results and with further investigation could lead to an applicable method.

## Data Availability Statement

The original contributions presented in the study are included in the article/Supplementary Material, further inquiries can be directed to the corresponding author/s.

## Author Contributions

AM: investigation, validation, visualization, and writing-original draft preparation. GN: investigation. GS: investigation. IV: conceptualization, methodology, visualization, project administration, supervision, and writing – review and editing. All authors contributed to the article and approved the submitted version.

## Conflict of Interest

The authors declare that the research was conducted in the absence of any commercial or financial relationships that could be construed as a potential conflict of interest.
