# Supplementary material for: Improving Biological Treatment of Real Bilge Wastewater With Zero Valent Iron and Activated Charcoal Addition
Source: Front Bioeng Biotechnol. 2020 Dec 18;8:614510. doi: 10.3389/fbioe.2020.614510 (PMC7775477; doi:10.3389/fbioe.2020.614510)
Supplement: Supplementary file 1 [file Table_1.DOCX]

**Supplementary material for the article:**

Improving biological treatment of real bilge wastewater with zero valent iron and activated charcoal addition

Aikaterini A. Mazioti^1^, Gregoris Notarides^1^, Giannis Symeou^1^, Ioannis Vyrides^1^*

^1^Environmental Engineering Laboratory, Department of Chemical Engineering, Cyprus University of Technology, 30 Archibishop Kyprianos str. 3036, Limassol, Cyprus

* Correspondence: Dr. Ioannis Vyrides, ioannis.vyrides@cut.ac.cy

|   a) |
| --- |
|   b) |
|   c) |

**Figure S1:** Treatment of real bilge wastewater with anaerobic granular sludge, with and without the simultaneously addition of ZVI and activated charcoal (AC); a) CH_4_% in headspace over time, b) CH_4_ cumulative production (ml) over time, c) COD values (mg L^-1^) of the wastewater over treatment time.
